# Oncologist perspectives on chemotherapy‐induced nausea and vomiting (CINV) management and outcomes: A quantitative market research‐based survey

**DOI:** 10.1002/cnr2.1127

**Published:** 2018-10-09

**Authors:** Matti Aapro, Pierfrancesco Ruffo, Roger Panteri, Stefano Costa, Vittoria Piovesana

**Affiliations:** ^1^ Genolier Cancer Centre, Clinique de Genolier Genolier Switzerland; ^2^ Department of Market Access, Helsinn Healthcare SA Pazzallo (Lugano) Switzerland; ^3^ Genactis Italia srl Rome Italy

**Keywords:** adherence, antiemetic therapy, chemotherapy‐induced nausea and vomiting (CINV), compliance, guidelines

## Abstract

**Background:**

Chemotherapy‐induced nausea and vomiting (CINV) is a distressing side effect that can negatively impact patients' quality of life and could discourage completion of chemotherapy, thereby affecting overall treatment outcomes. Although adherence to antiemetic guidelines can reduce CINV incidence in patients receiving highly or moderately emetogenic chemotherapy, CINV control remains inadequate.

**Aims:**

The objectives of this survey were to determine oncologists' practice patterns in CINV management, identify factors that contribute to antiemetic treatment failure, and determine the outcomes of uncontrolled CINV on health care resource utilisation and on patients' attitude towards chemotherapy.

**Methods and results:**

Quantitative market research was performed using an online questionnaire. Responses from 300 European oncologists who prescribe antiemetics and see ≥50 patients/month were analysed. Results showed that the main reasons reported by oncologists for antiemetic treatment failure were underestimating the emetogenic potential of chemotherapy, utilising weaker antiemetic regimens than required, and patient non‐adherence because of administration mistakes or missed/delayed doses. Educational initiatives for the oncology multidisciplinary team may help improve guideline‐consistent prescribing. Also, the availability of simpler, more convenient antiemetic therapies may improve guideline adherence and patient compliance during home administration.

**Conclusion:**

Achieving effective CINV control is a crucial goal to improve patients' quality of life, which should optimise chemotherapy outcomes, and would ultimately reduce health care costs.

## 1 INTRODUCTION

Chemotherapy‐induced nausea and vomiting (CINV), and nausea in particular, remain amongst the most dreaded and distressing side effects of anticancer treatment, negatively impacting patients' quality of life as well as affecting day‐to‐day functioning and nutritional status.^1^, ^2^ CINV can discourage patients from completing planned chemotherapy regimens, which may ultimately impact clinical outcomes.^3^ In a recent study, a direct correlation between the use of antiemetics and chemotherapy treatment compliance was demonstrated, where the use of the 5‐hydroxytryptamine‐3 receptor antagonist (5‐HT_3_ RA) palonosetron was shown to improve adherence to highly emetogenic chemotherapy (HEC) or moderately EC (MEC) regimens.^3^ Therefore, the optimal control of CINV with proper selection of antiemetics is a key factor in ensuring the completion of chemotherapy.

Current recommendations for prevention of CINV in patients receiving HEC or anthracycline‐cyclophosphamide–based chemotherapy, issued by the Multinational Association of Supportive Care in Cancer and the European Society for Medical Oncology (MASCC/ESMO), include the combination of a neurokinin‐1 (NK_1_) RA, a 5‐HT_3_ RA, and dexamethasone, amongst others,^4^ while the American Society of Clinical Oncology (ASCO) and the National Comprehensive Cancer Network (NCCN) guidelines recommend a four‐drug combination, with addition of olanzapine to the triplet.^5^, ^6^ The NK_1_ RA–5‐HT_3_ RA–dexamethasone combination is also advised for patients receiving carboplatin (at any dose)‐based chemotherapy,^4^ while ASCO and NCCN guidelines recommend the triplet combination only when carboplatin is administered at an area under the curve of 4.^5^, ^6^ For CINV prevention in patients receiving MEC, guidelines recommend a 5‐HT_3_ RA plus dexamethasone,^4^, ^5^, ^6^ and the NK_1_ RA–5‐HT_3_ RA–dexamethasone combination in selected patients with high‐risk factors for CINV, or for whom previous treatment with 5‐HT_3_ RA plus dexamethasone has failed.^5^

Effective prevention of CINV in the first 24 hours after chemotherapy (acute CINV) is critical to reduce its incidence in subsequent days (days 2‐5, delayed CINV).^7^ Additionally, it has been shown that effective CINV control during cycle 1 of chemotherapy is important to reduce the risk of CINV in subsequent cycles, and to reduce anticipatory nausea, a challenging symptom that involves anxiety and psychological factors relating to previous experience of CINV.^8^, ^9^ These studies demonstrate that early control of CINV is vital for optimal CINV management throughout the entire chemotherapy schedule. However, despite advances in antiemetic therapy, a significant proportion of patients receiving chemotherapy outside of randomised clinical trials still suffer from nausea and vomiting, which may indicate suboptimal use of evidence‐based antiemetic therapy guidelines in clinical practice.^3^, ^10^, ^11^ In fact, while guideline‐consistent antiemetic therapy has been shown to improve CINV control in cancer patients, there appear to be barriers to the use of these guidelines by health care professionals.^10^, ^11^, ^12^

Low adherence to use of antiemetics by patients at home may also contribute to the suboptimal management of CINV, since poor compliance to treatment is fairly common in many diseases and correlates with poorer outcomes and increased health care costs. The reasons for low adherence are often complex and include patient characteristics as well as the nature of the treatment regimen.^13^ The growing use of oral chemotherapy and supportive medications administered at home increases the potential for non‐adherence by patients, with multiple consequences, including unnecessary therapy adjustments because of a perceived lack of response, increased health care costs, and increased toxicities if the medication is not taken as prescribed.^13^

Hence, the effectiveness of antiemetic therapy in preventing CINV relies on the efficacy of antiemetic agents, physicians prescribing in accordance with treatment guidelines, and patients adhering to the treatment regimen. Identifying the barriers to utilising guideline‐recommended antiemetics in clinical practice may help design more‐convenient antiemetic regimens that increase treatment adherence and ultimately improve clinical outcomes.

The objectives of this quantitative market research‐based survey were to determine oncologists' practice patterns in CINV management, identify factors that might contribute to antiemetic treatment failure, determine the outcomes of uncontrolled CINV on the use of health care resources, identify whether oncologists detect changes in the attitude of their patients towards the planned chemotherapy after experiencing CINV, and to recognise the consequences of non‐compliance with antiemetic guideline recommendations in the prescription patterns of oncologists.

## 2 METHODS

### 2.1 Survey design and inclusion criteria

Quantitative market research based on an online survey was performed in May 2012. The questionnaire was designed by Genactis Italy Srl and medical specialists at Helsinn Healthcare, and based on current literature and antiemetic guidelines at the time of study conduct. The survey setup and raw data collection were performed using a platform written in C#/.net, which integrates a MS‐SQL database, and is run over a secured multitier Web architecture. The questionnaire was programmed using the Questionnaire Markup Language, a high‐level semantic XML language. The 11 questions included in the survey are listed in Table 1.

**Table 1:** Survey questions

Question	Answers	
Are you aware of the following guidelines for the prescribing of antiemetic therapy?	ASCO MASCC NCCN	□ yes □ no □ yes □ no □ yes □ no
To what extent do you adhere to the following guidelines when prescribing antiemetics? Please indicate your level of adherence to these guidelines using a 1 to 7 scale where 1 = I don't adhere to this guideline and 7 = I completely adhere to this guideline.	ASCO MASCC NCCN	value 1–7 value 1–7 value 1–7
In the columns below, please indicate what percentages of your patients receive each of the indicated antiemetic drugs or drug combinations regardless of line of therapy or administration	Moderately emetogenic chemotherapy regimens	Highly emetogenic chemotherapy regimens
Steroids monotherapy (eg, dexamethasone monotherapy) 5‐HT_3_ antagonist monotherapy +/− steroids (eg, ondansetron [Zofran], granisetron [Kytril], tropisetron [Navoban] monotherapy) 5‐HT_3_ antagonist monotherapy +/− steroids (eg, palonosetron [Aloxi] monotherapy) NK_1_ antagonist monotherapy +/− steroids (eg. aprepitant [Emend] monotherapy) NK_1_ antagonist monotherapy +/− steroids (eg, fosaprepitant [Ivemend] monotherapy) 5‐HT_3_ antagonist + NK_1_ antagonist +/− steroids (eg, ondansetron/granisetron/tropisetron + aprepitant) 5‐HT_3_ antagonist + NK_1_ antagonist +/− steroids (eg, ondansetron/granisetron/tropisetron + fosaprepitant) 5‐HT_3_ antagonist + NK_1_ antagonist +/− steroids (eg, palonosetron + aprepitant) Other drug or drug combination—specify	% a % b % c % d % e % f % g % h % i	% a % b % c % d % e % f % g % h % i
In your personal clinical practice, how do you consider the following regimens in terms of emetogenic potential when it comes to decide for the antiemetic drugs? Cisplatin >50 mg/m^2^; cisplatin <50 mg/m^2^; cyclophosphamide >1500 mg/m^2^; cyclophosphamide <1500 mg/m^2^; anthracyclines + cyclophosphamide (AC)	mildly emetogenic moderately emetogenic highly emetogenic	
For the categories below, please indicate what percentages of your patients who receive antiemetic drugs report emesis, hence you consider as non‐responders to current antiemetic treatments.	MEC – Acute emesis MEC – Delayed emesis HEC – Acute emesis HEC – Delayed emesis	
Considering your patients who reported emesis despite antiemetic treatments what percentage experience nausea, or vomiting, or both?	patients reporting only nausea patients reporting only vomiting patients reporting both nausea and vomiting	
In your opinion, what are the main reasons why patients report emesis despite being treated? Please indicate what percentage of patients experience emesis for the following reasons in your personal practice:	actual emetogenicity higher than expected “weaker” antiemetics (eg, monotherapy instead of combination) were used mistakes/issues with the administration (ie, time of administration, etc) other: Mainly psychological cofactors, anxiety, individual sensitivity	
Considering all your patients treated with antiemetic therapies for whom you prescribe treatments to take at home, what percentage of these patients made mistakes/missed one or more administrations?	Please indicate the percentage of patients	
What percentage of your patients who receive chemotherapy treatment or target therapy undergo additional medical visits or require additional therapy (eg, you had to undertake an unplanned visit and/or prescribe a rescue antiemetic treatment) for emesis‐related reasons after receiving their cycle of chemotherapy?	After MEC >30% 21%‐30% 11%‐20% 1%‐10% None	After HEC >30% 21%‐30% 11%‐20% 1%‐10% None
To what extent do you perceive unplanned visits and/or changes in planned antiemetic treatment due to emesis problems in treated patients as an issue in your personal clinical practice? Please answer using a 1 to 7 scale, where 1 = it is not at all an issue and 7 = it is a major issue.	1 2 3 4 5 6 7	
To what extent do you perceive hospitalisation due to emesis as an issue in your personal clinical practice. Please answer using a 1 to 7 scale where 1 = it is not at all an issue and 7 = it is a major issue.	1 2 3 4 5 6 7	
To what extent do you perceive patient adherence/compliance to antiemetic treatments as an issue in your personal clinical practice? Please answer using a 1 to 7 scale, where 1 = it is not at all an issue and 7 = it is a major issue.	1 2 3 4 5 6 7	
Please indicate how much you agree with the list of statements below, indicating 7 for agree completely and 1 for disagree completely. A I sometimes avoid or reduce highly emetogenic chemotherapy for some patients because of chemotherapy‐induced nausea and vomiting B Patients sometimes ask to change or cancel chemotherapy because they experienced emesis on previous courses of therapy C An antiemetic drug administered orally even on day 1 would be much appreciated by me and my patients	1 (disagree completely) 2 3 4 5 6 7 (agree completely)	

Abbreviations: ASCO, American Society of Clinical Oncology; HEC, highly emetogenic chemotherapy; MASCC, Multinational Association of Supportive Care in Cancer; MEC, moderately emetogenic chemotherapy; NCCN, National Comprehensive Cancer Network.

Survey participants met the following criteria: lived in Italy, France, Germany, Spain, or the UK; were registered oncologists; were common prescribers of antiemetic therapies; and at the time of the survey treated an average of at least 50 cancer patients per month. Potential participants were sent an email invitation that contained a direct link to the Web site hosting the survey. Upon accessing the Web site, participants were provided with a short description of the study and were asked to accept a confidentiality agreement before entering the survey. Participants then answered a screening question regarding the average number of cancer patients they see in a month (for all); participants from Germany were also asked to indicate their type of practice (office/private practice or hospital practice) and were eligible to participate up until meeting a final allocation target of 50% in office practice and 50% in hospital practice. Only respondents who met the eligibility criteria and passed the screening were allowed to proceed to the survey, which was estimated to be completed in approximately 20 minutes.

Participants were blinded to the study sponsor. Responses were based on oncologists' perceptions at the time of survey completion and did not involve the review of patients' files. Individual patient data were not collected; therefore, institutional review board assessment was not required.

### 2.2 Statistical analyses

Data from the survey were summarised by descriptive statistics. The frequencies (percentage) were calculated where applicable. The compiled data collected from participants from the five European countries are presented (*n* = 299). In addition, the perceived frequency of non‐adherence to treatment during home administration of antiemetics is shown for the individual countries.

For the analysis of responses to survey question 4, the percentages of patients in therapy options b and c were combined in the “5‐HT_3_ RA +/– steroids” group, the percentages of patients in therapy options d and e were combined in the “NK_1_ RA +/– steroids” group, and the percentages of patients in therapy options f, g, and h were combined in the “5‐HT_3_ RA + NK_1_ RA +/– steroids” group.

In the analysis of responses, scores were grouped into the following predetermined categories

- Minor/no issue (scores 1‐3 of a 1‐7 scale, where 1 = it is not at all an issue and 7 = it is a major issue);
- Moderate/major issue (scores 5‐7 of a 1‐7 scale, where 1 = it is not at all an issue and 7 = it is a major issue);
- Low/no agreement (scores 1‐3 of a 1‐7 scale, where 1 = disagree completely and 7 = agree completely);
- Moderate/high agreement (scores 5‐7 of a 1‐7 scale, where 1 = disagree completely and 7 = agree completely.

## 3 RESULTS

### 3.1 Survey participants

**Figure 1:**
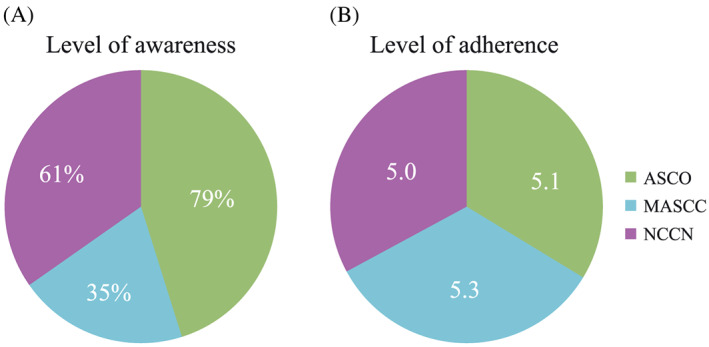
Level of awareness and use of international antiemetic guidelines. Respondent rates^†^ to (A) question 1^‡^ and (B) question 2.^§^ ASCO, American Society of Clinical Oncology; MASCC, Multinational Association of Supportive Care in Cancer; NCCN, National Comprehensive Cancer Network. ^†^Combined responses from oncologists from Italy, France, Germany, Spain, and the United Kingdom. ^‡^Question 1: Are you aware of the following guidelines for the prescribing of antiemetic therapy? ASCO/MASCC/NCCN. ^§^Question 2: To what extent do you adhere to the following guidelines when prescribing antiemetics? ASCO/MASCC/NCCN. Please indicate your level of adherence to these guidelines using a 1 to 7 scale where 1 = I don't adhere to this guideline and 7 = I completely adhere to this guideline

**Figure 2:**
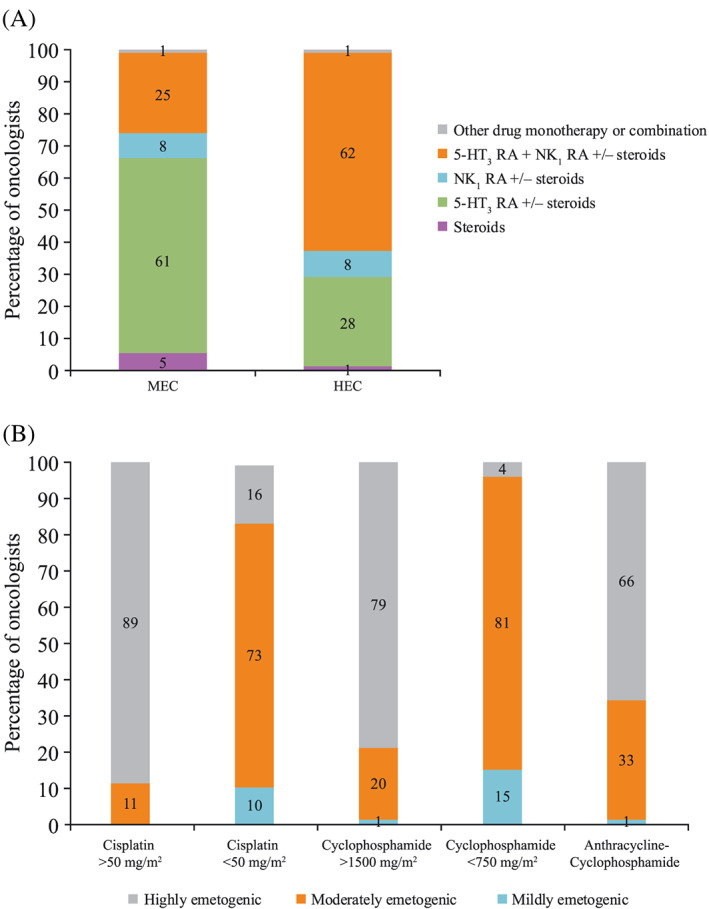
(A) Antiemetic prescription patterns for the prevention of CINV associated with MEC and HEC, and (B) emetogenic risk of chemotherapeutic regimens as perceived by oncologists. Respondent rates^†^ to (A) question 3^‡^ and (B) question 4.^§^ 5‐HT_3_ RA, 5‐hydroxytryptamine‐3 receptor antagonist; CINV, chemotherapy‐induced nausea and vomiting; HEC, highly emetogenic chemotherapy; MEC, moderately emetogenic chemotherapy; NK_1_, neurokinin 1. ^†^Combined responses from oncologists from Italy, France, Germany, Spain, and the United Kingdom. ^‡^Question 3: Please indicate what percentages of your patients receive each of the indicated antiemetic drugs or drug combinations regardless of line of therapy or administration: *Steroids monotherapy; 5‐HT*_*3*_ *RA monotherapy +/− steroids; NK*_*1*_ *RA monotherapy +/− steroids; 5‐HT*_*3*_ *RA + NK*_*1*_ *RA +/− steroids; Other drug or drug combination—specify*. ^§^Question 4: In your personal clinical practice, how do you consider the following regimens in terms of emetogenic potential when it comes to decide for the antiemetic drugs? Cisplatin >50 mg/m^2^; cisplatin <50 mg/m^2^; cyclophosphamide >1500 mg/m^2^; cyclophosphamide <1500 mg/m^2^; anthracyclines + cyclophosphamide (AC). For each regimen, indicate: *mildly emetogenic; moderately emetogenic; highly emetogenic*

**Figure 3:**
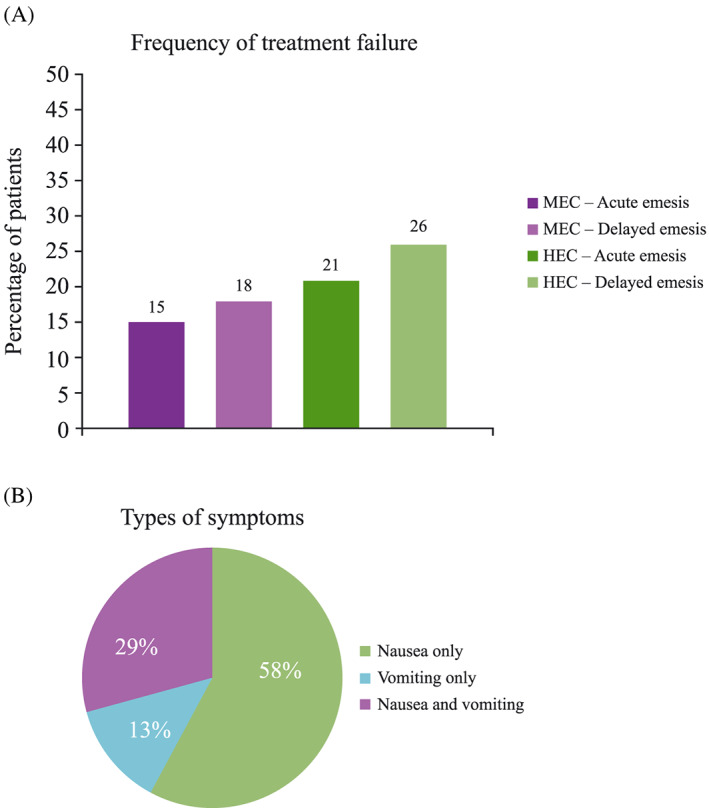
(A) Incidence of uncontrolled CINV in patients who receive antiemetic drugs prior to HEC and MEC during the acute and delayed phases, and (B) percentage of patients experiencing nausea only, vomiting only, or both. Respondent rates^†^ to (A) question 5^‡^ and (B) question 6.^§^ HEC, highly emetogenic chemotherapy; MEC, moderately emetogenic chemotherapy. ^†^Combined responses from oncologists from Italy, France, Germany, Spain, and the United Kingdom. ^‡^Question 5: For the categories below, please indicate what percentages of your patients who receive antiemetic drugs report emesis, hence you consider as non‐responders to current antiemetic treatments: *MEC—Acute emesis; MEC—Delayed emesis; HEC—Acute emesis; HEC—Delayed emesis*. ^§^Question 6: Considering your patients who reported emesis despite antiemetic treatments, what percentage experience nausea, or vomiting, or both? *Patients reporting only nausea; Patients reporting only vomiting; Patients reporting both nausea and vomiting*

**Figure 4:**
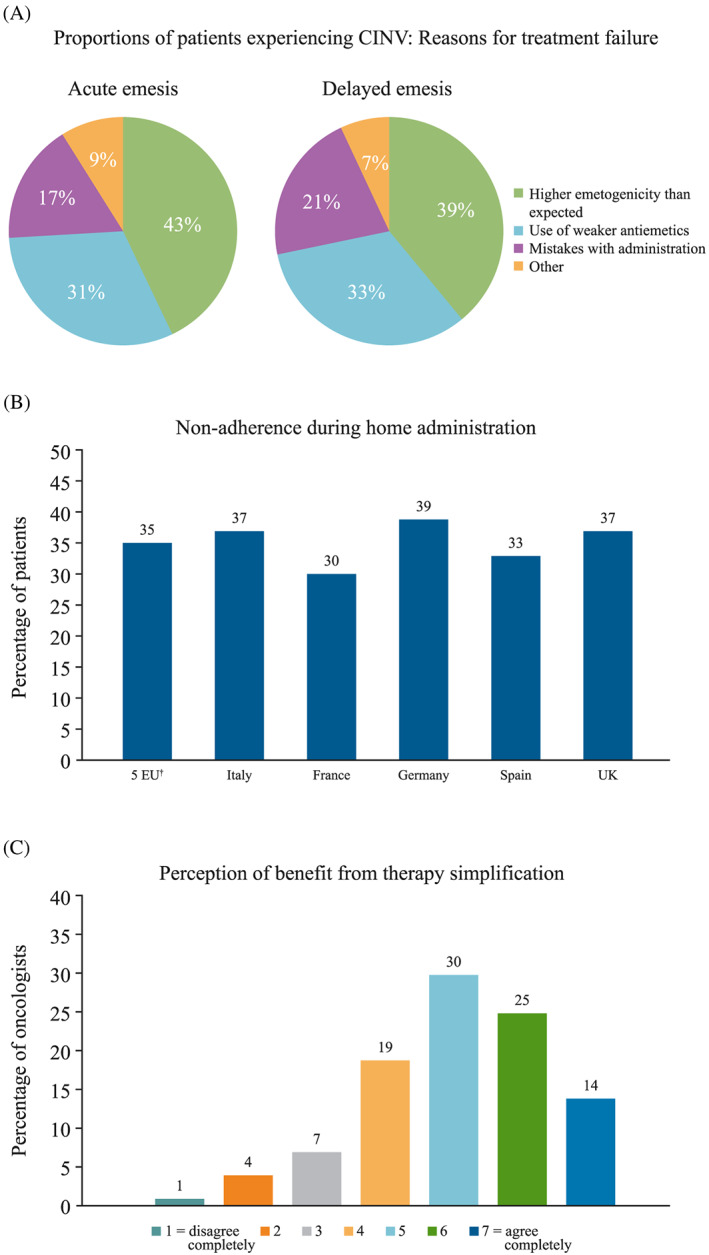
(A) Reasons for experiencing acute and delayed emesis, (B) frequency of non‐adherence to antiemetic treatment by patients, and (C) oncologists' perceptions about the benefit of patient adherence to simplified antiemetic treatments. Respondent rates^†^ to (A) question 7^‡^, (B) question 8^§^, and (C) question 13C.^¶^ CINV, chemotherapy‐induced nausea and vomiting; UK, United Kingdom. ^†^Combined responses from oncologists from Italy, France, Germany, Spain, and the United Kingdom. ^‡^Question 7: In your opinion, what are the main reasons why patients report emesis despite being treated? Please indicate what percentage of patients experience emesis for the following reasons in your personal practice. (Separate questions were asked for acute and delayed emesis.). *Actual emetogenicity higher than expected; “Weaker” antiemetics (eg, monotherapy instead of combination) were used; Mistakes/issues with the administration (ie, time of administration, etc); Other: mainly psychological cofactors, anxiety, individual sensitivity*. ^§^Question 8: Considering all your patients treated with antiemetic therapies for whom you prescribe treatments to take at home, what percentage of these patients made mistakes/missed 1 or more administrations? Please indicate the percentage of patients. ^¶^Question 13C: Please indicate how much you agree with the statement below, indicating 7 for agree completely and 1 for disagree completely. *“An antiemetic drug administered orally even on day 1 would be much appreciated by me and my patients”*

**Figure 5:**
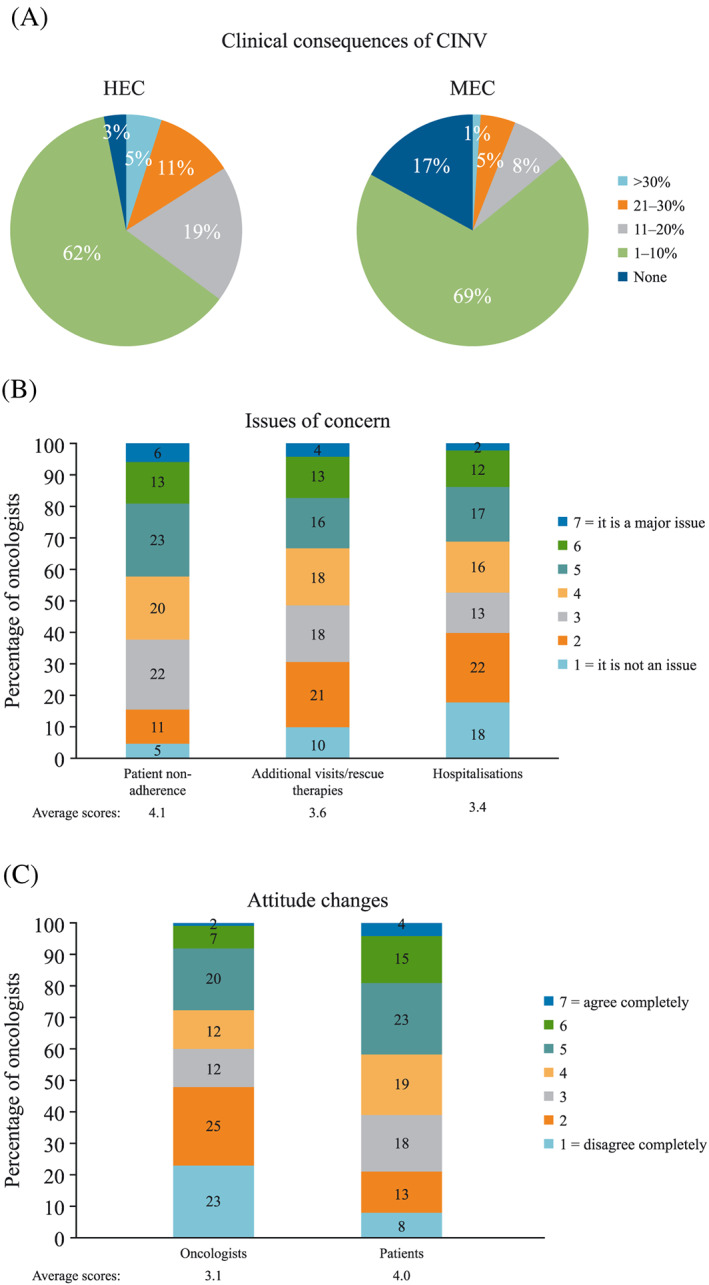
(A) Frequency of patients requiring additional medical visits or rescue therapy because of uncontrolled CINV. (B) Degree of concern amongst oncologists about the clinical consequences of uncontrolled CINV. (C) Changes in the attitudes of oncologists and patients after uncontrolled CINV. Respondent rates^†^ to (A) question 9^‡^, (B) questions 10, 11, and 12^§^, and (C) questions 13^¶^ A and B. CINV, chemotherapy‐induced nausea and vomiting; HEC, highly emetogenic chemotherapy; MEC, moderately emetogenic chemotherapy. ^†^Combined responses from oncologists from Italy, France, Germany, Spain, and the United Kingdom. ^‡^Question 9: What percentage of your patients who receive chemotherapy treatment or target therapy undergo additional medical visits or require additional therapy (eg, you had to undertake an unplanned visit and/or prescribe a rescue antiemetic treatment) for emesis‐related reasons after receiving their cycle of chemotherapy? (Separate questions were asked for MEC and HEC.). *>30%; 21% to 30%; 11% to 20%; 1% to 10%; None*. ^§^Question 10: To what extent do you perceive unplanned visits and/or changes in planned antiemetic treatment due to emesis problems in treated patients as an issue in your personal clinical practice? Question 11: To what extent do you perceive hospitalisation due to emesis as an issue in your personal clinical practice?Question 12: To what extent do you perceive patient adherence/compliance to antiemetic treatments as an issue in your personal clinical practice? Please answer using a 1 to 7 scale, where 1 = it is not at all an issue and 7 = it is a major issue. ^¶^Question 13: Please indicate how much you agree with the list of statements below, indicating 7 for agree completely and 1 for disagree completely. *A. I sometimes avoid or reduce highly emetogenic chemotherapy for some patients because of chemotherapy‐induced nausea and vomiting*. *B. Patients sometimes ask to change or cancel chemotherapy because they experienced emesis on previous courses of therapy*

One thousand four hundred fifty‐five oncologists were invited to participate in the survey, of whom a total of 299 responded and completed the survey (1141 oncologists did not access or did not complete the questionnaire, and 15 oncologists were screened out). Overall, 60 oncologists each from Italy, France, Germany, and Spain, and 59 oncologists from the UK participated in the survey. All of the respondents from France, Italy, Spain, and the UK were hospital oncologists. Amongst the German participants, 50% were hospital oncologists and 50% were office‐based oncologists. Responses from participating oncologists were combined and are presented in Figures 1, 2, 3, 4, 5.

### 3.2 Degree of awareness and adherence to international antiemetic guidelines

Amongst the participating oncologists, awareness of the ASCO antiemetic guidelines was highest, followed by the NCCN and MASCC/ESMO guidelines (Figure 1A).

Overall, a moderate to high degree of adherence to guidelines was reported (Figure 1B), with a similar perceived level of adherence for the three guidelines (5.0‐5.3, scale 1‐7, where 7 = complete adherence).

### 3.3 Antiemetics prescription patterns and perceived emetogenic potential of chemotherapy

At the time the survey was performed, antiemetic recommendations from ASCO, NCCN, and MASCC/ESMO guidelines included the use of a 5‐HT_3_ RA + dexamethasone for patients receiving MEC, and the NK_1_ RA + 5‐HT_3_ RA + dexamethasone triplet combination for patients receiving HEC. Oncologist‐reported antiemetics prescription in clinical practice shows a maximum adherence rate of 61% and 62% for patients treated with MEC and HEC, respectively (Figure 2A). These adherence rates most probably represent an overestimation, since it is likely that not all patients received dexamethasone as part of their prophylactic regimen.

In general, the emetogenic potential of chemotherapy was underestimated, with 11%, 83%, and 21% of oncologists perceiving cisplatin (>50 mg/m^2^), cisplatin (<50 mg/m^2^), and cyclophosphamide (>1500 mg/m^2^), respectively, as mildly or moderately emetogenic. One third of respondents considered anthracycline‐cyclophosphamide regimens (currently classified as HEC) to be moderately emetogenic (Figure 2B).

### 3.4 Perceived incidence of CINV and types of symptoms with current antiemetic therapies

Despite antiemetic prophylaxis, respondents reported an incidence of CINV of 15% (acute phase) and 18% (delayed phase) in their patients receiving MEC, and 21% (acute phase) and 26% (delayed phase) in patients receiving HEC (Figure 3A). Of those patients experiencing CINV, oncologists reported that most patients experience nausea only (58%), approximately a third of patients experience both nausea and vomiting (29%), and 13% of patients experience vomiting only (Figure 3B).

### 3.5 Potential reasons for antiemetic treatment failure

The estimated proportions of patients experiencing CINV because of antiemetic treatment failure for various reasons are shown in Figure 4A. The main reason cited for treatment failure during the acute and delayed phases was that actual chemotherapy emetogenicity was higher than expected (43% and 39% for acute and delayed CINV, respectively). The second most important reason cited by the survey participants was the use of “weaker” antiemetic regimens than required, such as use of monotherapy instead of combinations, which results in emesis in approximately a third of patients during both the acute and delayed phases (31% and 33%, respectively). An additional concern for oncologists was errors during the administration of antiemetics, including mistakes or issues with administration, which were perceived as a reason for treatment failure affecting more patients in the delayed phase (21% vs 17% in the acute phase). Oncologists from all European countries consistently reported that during home administration of antiemetics, approximately a third of patients (range: 30%‐39%) made administration mistakes or missed/delayed one or more doses (Figure 4B). This non‐adherence to antiemetic treatment by patients was perceived as a moderate/major issue by nearly half of oncologists (42%) (Figure 5B). The potential benefit of antiemetic therapy simplification as a means to improve CINV control was explored, and most oncologists (69%) thought that an antiemetic drug administered orally on day 1 would be appreciated in their clinical practice and by their patients (Figure 4C).

### 3.6 Potential consequences of antiemetic treatment failure

A total of 35% and 14% of oncologists considered that >10% of patients undergo additional medical visits or require additional antiemetic therapy for CINV‐related reasons after HEC and MEC, respectively (Figure 5A). Approximately one‐third of respondents (33%) perceived this need for additional visits or for rescue antiemetic therapies as an issue of moderate to major concern (Figure 5B). Similarly, one‐third of respondents (31%) perceived hospitalisation because of emesis as an important issue (Figure 5B). The failure of antiemetic treatment led to changes in attitude towards the design of the antiemetic regimen for physicians, and towards the planned chemotherapy treatment for patients (Figure 5C), with approximately a third of oncologists (29%) reporting that they agreed that they sometimes avoid or reduce HEC for some patients because of CINV. A total of 42% of oncologists agreed that patients sometimes ask to change or cancel chemotherapy because of previous CINV episodes.

## 4 DISCUSSION

The findings from this survey reveal that while the participating oncologists reported high awareness and adherence to antiemetic guidelines, a number of patients still do not respond to antiemetic treatments and suffer debilitating CINV. The main reasons reported for antiemetic treatment failure were underestimating the emetogenic potential of chemotherapy and utilising weaker antiemetic regimens than required. In line with this, a substantial proportion of respondents perceived cisplatin, cyclophosphamide >1500 mg/m^2^, and the anthracycline‐cyclophosphamide regimen as mildly or moderately emetogenic. Additionally, approximately one‐third of oncologists indicated that they prescribe either an NK_1_ RA or a 5‐HT_3_ RA in monotherapy (with or without steroids) for antiemetic prophylaxis in HEC‐treated patients. The third reason reported for treatment failure was non‐adherence with treatments because of mistakes in administration by patients, which were estimated to occur in approximately a third of patients during home administration of antiemetics. However, these reasons somewhat contradict the reportedly perceived high awareness and adherence to the guidelines by the participants. The survey also highlighted oncologists' perception that the effects of antiemetic treatment failure led to increased unplanned hospital visits, hospitalisations, and the use of rescue medication. Experiencing antiemetic failure was also accompanied by a shift in attitude in both physicians and patients, leading to modifications in prescribing antiemetics by oncologists and requests for changes in chemotherapy by patients.

A potential cause of antiemetic treatment failure may be associated with the administration of antiemetics that is inconsistent with guideline recommendations. In this survey, a high level of adherence to antiemetic guidelines was reported by participating oncologists. However, the reported prescription patterns (Figure 2A) showed suboptimal adherence to guidelines, which is in line with observations in more‐recent studies.^10^, ^11^, ^14^, ^15^, ^16^ In addition, recent surveys assessing perceptions and practice patterns amongst oncology nurses in the US^12^ and Europe^17^ revealed that, from the nurses' perspective, physician preference is the main barrier to guideline‐recommended prescription of antiemetic prophylaxis.^14^

Consistent with the results from the present survey, various studies have shown that the incidence of CINV is often underestimated by medical oncologists and oncology nurses,^18^ especially during the delayed phase, after both MEC and HEC.^19^, ^20^ Several patient‐related factors that increase the likelihood of emesis have been identified, including younger age, female gender, low alcohol intake, anxiety, and history of motion sickness or nausea during pregnancy.^21^, ^22^ However, patient‐related risk factors are usually not considered when selecting antiemetic treatment. To assist physicians in this area, the MASCC Antiemesis Tool validated by Dr Molassiotis and colleagues^23^ (available at: http://www.mascc.org) and the prediction tool developed by Dranitsaris and colleagues^24^ (available at http://cinvrisk.org) can provide valuable assistance with the evaluation of patient‐related risk factors when making treatment decisions. Integration of the patient's personal risk factors will allow for more efficient control of nausea and vomiting and optimise antiemetic use.^16^ In addition, re‐evaluation of chemotherapeutic agents and/or regimens in specific patient populations may be of value in uncovering the need for a triplet antiemetic combination in particular settings.

Non‐adherence during home administration of antiemetics was identified as an issue in approximately one‐third of patients, which supports the results of the recent surveys of oncology nurses, where patients' non‐adherence to treatment was ranked amongst the top challenges in managing CINV.^12^, ^17^ Non‐adherence can lead to worse outcomes of chemotherapy, as reported in patients with breast cancer receiving anthracycline‐based chemotherapy.^25^ While there can be multiple reasons for non‐adherence to antiemetic therapy, patient characteristics, regimen complexity, and education about antiemetics are important factors. Additionally, in a recent study, some patients specified that they waited until they felt nauseated before taking the medication, failing to understand that antiemetics are taken for the prophylaxis of CINV.^18^ Therefore, numerous strategies that involve a multidisciplinary team of health care professionals and include active educational initiatives, as well as highlighting the importance of patient feedback on outcomes, are vital to improve guideline adherence by physicians and adherence to antiemetic treatment by patients.^14^

An additional strategy to improve CINV control may be the simplification of antiemetic treatments. Ultimately, this may lead to improved adherence and ensure chemotherapy completion. A number of studies have shown that reducing pill burden and using fixed‐dose combination agents can improve treatment adherence by patients in various diseases, including human immunodeficiency virus, tuberculosis, and hypertension.^26^, ^27^, ^28^

The NK_1_ RAs aprepitant, fosaprepitant, rolapitant, and NEPA, the oral fixed‐combination antiemetic agent (composed of the NK_1_ RA netupitant and the 5‐HT_3_ RA palonosetron), have demonstrated high efficacy and safety for the control of CINV. These agents are now included in the antiemetic regimens recommended by international antiemetic guidelines for CINV prophylaxis.^4^, ^5^, ^6^ The various regimens differ in complexity depending on the route of administration, number of pills, and days of treatment. All NK_1_ RAs are administered prior to chemotherapy. Due to their prolonged half‐lives, fosaprepitant, NEPA, and rolapitant are only administered on the day of chemotherapy. NEPA is administered as a single dose, ensuring the correct administration of the NK_1_ and 5‐HT_3_ RAs under the supervision of health care professionals. As the only fixed combination antiemetic, NEPA does not require the separate administration of a 5‐HT_3_ RA, reducing the need for follow‐up antiemetics at home and thereby facilitating adherence to treatment.^15^, ^29^ Dexamethasone is administered concomitantly with all NK_1_ RAs and 5‐HT_3_ RAs, with a similar recommended schedule of dosing. However, the dose of dexamethasone varies depending on the NK_1_ RA used in the combination and is reduced when co‐administered with aprepitant and NEPA due to drug‐drug interactions. Overall, NEPA requires a lower number of doses of antiemetic drugs to be administered during days 1 to 4 after chemotherapy, simplifying the antiemetic regimen.^15^ Amongst 5‐HT_3_ RAs, regimens that include intravenous palonosetron, and granisetron extended‐release injection or transdermal patch are also convenient options.

Improving the effectiveness of antiemetic therapies requires an understanding of their impact on current CINV management in a practical, real‐life setting. Clinical trials, performed under ideal conditions, in homogeneous patient populations, and where patients are closely monitored, are limited in their ability to provide a real indication of effectiveness.^30^ The strengths of this study include the high number of survey participants, comprising a good representation of oncologists from five European countries who commonly prescribe antiemetic therapies and treat an average of at least 50 cancer patients per month. Therefore, the outcomes can be considered as representing the “real world” and are valuable in improving understanding of the factors that impact the prescribing of antiemetic agents, and the effectiveness of therapy. The insights gained from the survey may also assist with development of educational initiatives to improve the uptake of guidelines, to develop new treatment schedules, and to recognise the importance of including patient‐reported outcomes in the antiemetic treatment decision process. Conversely, a limitation of this study is that since it was carried out in 2012, there have been significant developments in the field, with more antiemetic options available and updated evidence‐based guidelines released to assist health care professionals. However, the key findings of the survey are still relevant to current challenges in managing CINV. An additional limitation is the subjective nature of the survey, with bias in the responses (eg, the reported incidence of CINV and the perceived level of non‐adherence to antiemetic guidelines are likely to be underestimated).

In conclusion, the results from this survey emphasise the utmost importance of effective knowledge of antiemetic treatment guidelines and highlight the need to ensure that oncologists are aware of, understand, and follow published antiemetic guidelines. While treatment adherence was raised as an important issue by the survey respondents, the availability of (1) new fixed‐combination antiemetic treatment options, such as NEPA; (2) antiemetics with prolonged half‐lives, such as rolapitant and NEPA or single‐injection fosaprepitant amongst NK_1_ RAs, and intravenous palonosetron, granisetron extended‐release injection, or granisetron transdermal patch amongst 5‐HT_3_ RAs; and (3) intravenous formulation options of current antiemetics will allow for easier and more convenient administration of antiemetics, and may positively impact compliance by reducing the need for more‐frequent self‐administration at home for some agents. The convenience in administration might have greater impact in the real world, compared with what has been observed in clinical trials, where patients are closely monitored and adherence to treatment is likely to be higher. Finally, the results from this survey facilitate establishing interventions that can be put in place for the effective dissemination of antiemetic treatment guidelines, and educational initiatives that emphasise the importance of guideline‐consistent prescribing and of patient‐reported outcomes, in order to improve patients' quality of life, chemotherapy outcomes, and ultimately reduce health care costs.

## CONFLICT OF INTEREST DISCLOSURES

M. Aapro: advisor for Eisai, Helsinn, Merck, Mundipharma, Roche, and Tesaro; honoraria from Eisai, Helsinn, Merck, Mundipharma, Roche, and Tesaro; and has received grants from Helsinn, Merck, Roche, and Tesaro.

P. Ruffo: Helsinn Healthcare SA employee.

R. Panteri: no conflicts of interest.

S. Costa: no conflicts of interest.

V. Piovesana: Helsinn Healthcare SA employee.

## AUTHORS' CONTRIBUTIONS

All authors had full access to the data in the study and take responsibility for the integrity of the data and the accuracy of the data analysis. *Conceptualization*, V.P., P.R.; *Methodology*, R.P., S.C.; *Investigation*, R.P., S.C.; *Formal Analysis*, R.P., S.C.; *Resources*, R.P., S.C.; *Writing ‐ Original Draft*, M.A., V.P., P.R., R.P., S.C.; *Writing ‐ Review & Editing*, M.A., V.P., P.R., R.P., S.C.; *Visualization*, M.A., P.R.; *Supervision*, V.P., P.R.; *Funding Acquisition*, V.P., P.R.

## ETHICAL APPROVAL

Ethical approval is not applicable.
